# SEX IN NURSING HOMES? A PRELIMINARY ANALYSIS OF INTERVIEWS WITH COMMUNITY-DWELLING ADULTS

**DOI:** 10.1093/geroni/igz038.3139

**Published:** 2019-11-08

**Authors:** Rachael Spalding, Emma Katz, Barry Edelstein

**Affiliations:** West Virginia University, Morgantown, West Virginia, United States

## Abstract

Most older adults living in long-term care settings (LTCs) indicate that expressing their sexuality is important to them (Doll, 2013). However, negative views of late-life sexuality persist in the United States (Robinson & Molzahn, 2007), particularly among nursing staff in LTCs. Staff often express discomfort regarding residents’ sexual lives (Bouman, Arcelus, & Benbow, 2007), despite the fact that LTCs are residents’ homes where private behaviors such as sexual activity might be expected to occur. Little is known about the general public’s attitudes towards sexual behaviors in LTCs. Attitudes of LTC residents’ family members is particularly important, as they are most likely to visit residents and to care about their quality of life, in turn informing facility policies and management. In this study, we took preliminary steps toward gathering this information by focusing on attitudes of community-dwelling adults. Using an iterative approach, we conducted semi-structured interviews with community-dwelling adults (n = 9; age range = 18 – 65 years) regarding their beliefs about romantic relationships and sexual behaviors among LTC residents. Major themes were identified through thematic content analysis. Participants indicated favorable attitudes towards residents’ sexual expression as a means of combatting loneliness and fostering emotional intimacy. Other themes included residents’ rights to privacy, potential risks of sexual behavior, and the need to consider how individuals may differ in their ability to consent to sexual activity. This data is intended to inform the development of a measurement tool for use with LTC residents’ family members.

